# Resting Regulatory CD4 T Cells: A Site of HIV Persistence in Patients on Long-Term Effective Antiretroviral Therapy

**DOI:** 10.1371/journal.pone.0003305

**Published:** 2008-10-01

**Authors:** Tu-Anh Tran, Marie-Ghislaine de Goër de Herve, Houria Hendel-Chavez, Bamory Dembele, Emilie Le Névot, Karim Abbed, Coralie Pallier, Cécile Goujard, Jacques Gasnault, Jean-François Delfraissy, Anne-Marie Balazuc, Yassine Taoufik

**Affiliations:** 1 INSERM U802, Université Paris 11, Le Kremlin Bicêtre, France; 2 Unité d'Immunologie Biologique, Assistance Publique-Hôpitaux de Paris, Hôpital Bicêtre, Le Kremlin-Bicêtre, France; 3 Service de Médecine Interne, Assistance Publique-Hôpitaux de Paris, Hôpital Bicêtre, Le Kremlin-Bicêtre, France; 4 Laboratoire de Virologie, Assistance Publique-Hôpitaux de Paris, Hôpital Bicêtre, Le Kremlin-Bicêtre, France; 5 Institut Pasteur, Paris, France; Institut Pasteur Korea, Republic of Korea

## Abstract

**Background:**

In HIV-infected patients on long-term HAART, virus persistence in resting long-lived CD4 T cells is a major barrier to curing the infection. Cell quiescence, by favouring HIV latency, reduces the risk of recognition and cell destruction by cytotoxic lymphocytes. Several cell-activation-based approaches have been proposed to disrupt cell quiescence and then virus latency, but these approaches have not eradicated the virus. CD4^+^CD25^+^ regulatory T cells (Tregs) are a CD4^+^ T-cell subset with particular activation properties. We investigated the role of these cells in virus persistence in patients on long-term HAART.

**Methodology/Principal Findings:**

We found evidence of infection of resting Tregs (HLADR^−^CD69^−^CD25^hi^FoxP3^+^CD4^+^ T cells) purified from patients on prolonged HAART. HIV DNA harbouring cells appear more abundant in the Treg subset than in non-Tregs. The half-life of the Treg reservoir was estimated at 20 months. Since Tregs from patients on prolonged HAART showed hyporesponsiveness to cell activation and inhibition of HIV-specific cytotoxic T lymphocyte-related functions upon activation, therapeutics targeting cell quiescence to induce virus expression may not be appropriate for purging the Treg reservoir.

**Conclusions:**

Our results identify Tregs as a particular compartment within the latent reservoir that may require a specific approach for its purging.

## Introduction

Although highly active antiretroviral therapy (HAART) generally suppresses HIV replication to undetectable plasma levels for prolonged periods of time, it fails to eradicate the virus. Interruption of HAART almost invariably leads to rebound viral replication. This raises the question of how non resistant, non defective HIV can persist during long-term HAART. This issue was resolved in part by the identification of a small, stable pool of resting CD4^+^ T cells latently infected by replication-competent HIV [1]–[3]. This reservoir is mainly composed of cells with a memory phenotype [2], [4], [5], of which a significant proportion are HIV-specific [6], [7]. Cell quiescence, by favouring HIV latency, reduces the risk of recognition by cytotoxic CD8^+^ T cells and the risk of host cell destruction by direct viral cytopathogenic effects.

CD4^+^CD25^+^ regulatory T cells (Tregs) are a CD4^+^ T-cell subset with particular activation properties [8], [9]. Forkhead transcription factor (*FoxP3*) gene expression is required for their development and function [10]. In vitro, these cells are unresponsive to conventional T-cell stimuli such as anti-CD3, but co-stimulation by CD28 cross-linking or with interleukin 2 (IL-2) may overcome this anergy at least in part [11], [12]. In humans and mice, these cells constitutively express CD25 and have suppressive effects on T and B cells [8], [13]. Multiorgan autoimmune disorders result when this population is removed in normal mice and when *FoxP3* is mutated in both humans and mice [10]. Tregs also inhibit CD4 and CD8 T cell immune responses to several pathogens, including *Leishmania*, hepatitis C virus, and HIV [14]–[21]. During SIV infection, they could be depleted from the intestinal lamina propria [22]. Human Treg cells can be infected by HIV and permit its replication [18], [23]. We investigated the role of these cells in HIV persistence in patients on long-term HAART.

## Results

**Figure 1:**
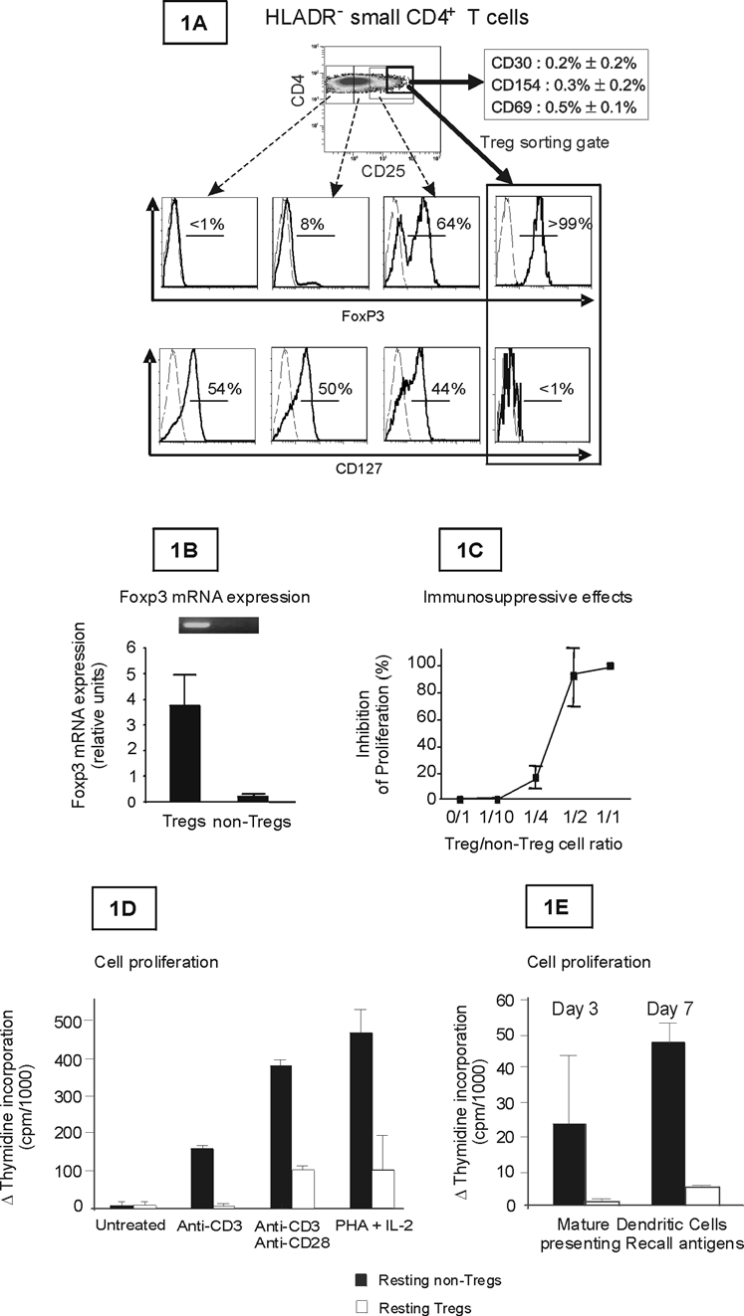
Definition of the resting Treg and non-Treg working cell populations. Fig. 1A: FoxP3 expression was examined by flow cytometry following cell permeabilization of PBMCs isolated from HIV-infected patients on long-term HAART (see Methods). FoxP3, CD25 and CD127 expression was analyzed in the HLADR^−^ CD4^+^ small lymphocyte gate. In Fig. 1B, mRNA was extracted from resting Tregs (CD25^hi^HLADR^−^ CD69^−^CD4^+^ small size T cells) and non-Tregs (CD25^−^HLADR^−^CD4^+^ small size T cells). Quantitative RT-PCR was then used to quantify FoxP3 and GAPDH cDNA. Results are expressed as relative units: FoxP3/GAPDH cDNA ratios (atograms/femtograms) and correspond to the mean±SEM of values obtained in 3 donors. Fig. 1C: Sorted resting non-Tregs (CD25^−^HLADR^−^CD4^+^small size T cells) were activated with plate-bound anti-CD3 mAb in the absence or presence of resting Tregs (CD25^hi^HLADR^−^ CD4^+^ small size T cells) (>99% FoxP3^+^) at ratios ranging from 1/10 to 1/1. Cell proliferation was measured. Results (mean±SEM, n = 2) were expressed as the percentage inhibition of proliferation compared to control cultures (without resting Tregs). Fig. 1D: 100 000 sorted CD25^−^HLADR^−^CD4^+^ T cells (black columns) or CD25^+^HLADR^−^CD4^+^T cells (white columns) (obtained as described in Methods) were cultured with plate-bound anti-CD3 mAb±soluble anti-CD28 mAb or with PHA+IL-2. Controls were untreated cells. In Fig. 1E, 100 000 freshly purified CD25^−^HLADR^−^CD4^+^ (black columns) or CD25^+^HLADR^−^CD4^+^ (white columns) T cells (see methods) were also co-cultured with 20 000 mature monocyte-derived DCs loaded in the immature state with a mix of recall antigens (Cytomegalovirus CMV, Purified Protein Derivative PPD, Tetanus toxoid TT and HIV p24). Controls were co-cultures of non-antigen-loaded mature DCs and T cells. Proliferation was evaluated on day 3 for anti-CD3±anti-CD28 activation and on days 3 and 7 for co-culture with mature DCs by measuring thymidine incorporation. Results are expressed as the difference between activated wells and control wells. The results are means±SEM for 4 patients.

Highly purified CD25^hi^HLADR^−^CD4^+^ small size T cells were obtained by cell sorting. For each patient tested, the CD25^hi^ sorting gate in HLADR^−^CD4^+^ small size lymphocytes was pre-defined on the basis of intracellular Foxp3 expression (>99% Foxp3^+^) (see Fig. 1A and methods). Expression of FoxP3 was confirmed by RT-PCR analysis (Fig. 1B) Expression of the activation markers CD30, intracellular CD40 ligand and CD69 in selected cells ranged from less than 0.1% to 0.6%. Cells were mainly CD127^low^ (Fig. 1A) as previously reported [24], [25] and suppressed conventional CD4 T-cell proliferation following polyclonal activation with anti-CD3 (Fig. 1C). Tregs also showed hyporesponsiveness following polyclonal activation or specific activation with recall antigens presented by mature dendritic cells (Fig. 1D and 1E). In subsequent virological studies, in addition to the markers described above, quiescent Tregs cells were also sorted on the basis of negative CD69 expression.

### Presence of an HIV reservoir in Tregs in patients on prolonged HAART

**Figure 2:**
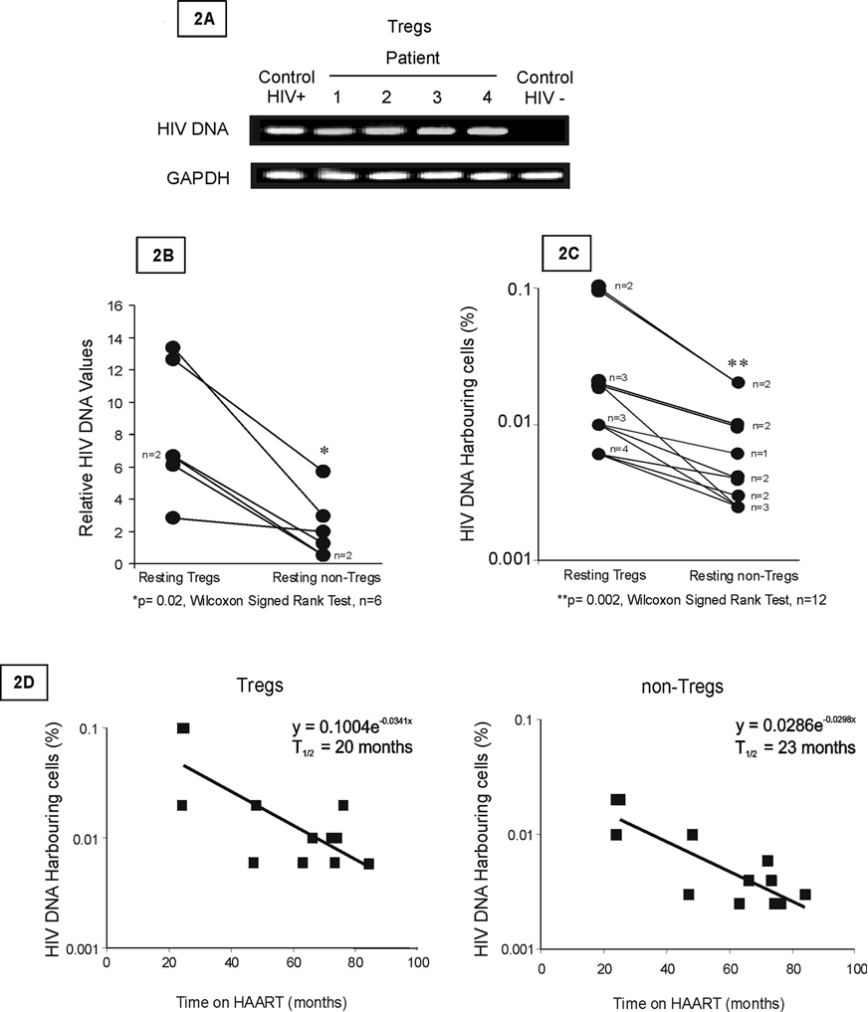
Presence of an HIV reservoir in resting Tregs in patients on prolonged HAART. Fig. 2A: DNA was extracted and analysed by PCR for HIV Env and GAPDH expression from resting Tregs (CD25^hi^HLADR^−^ CD69^−^CD4^+^ small size T cells) (>99% FoxP3^+^). Positive and negative controls were total CD4^+^ T cells from HIV^+^ and HIV^−^ individuals, respectively. Similar results were obtained in 4 other patients tested. Fig. 2B: quantitation of HIV DNA by real-time PCR as described in Methods, in resting Tregs and non-Tregs (CD25^−^HLADR^−^ CD69^−^CD4^+^ small size T cells). Results are expressed as atogramms of HIV DNA per 100 fentogramms of GAPDH. Results correspond to the mean±SEM of values obtained in 6 patients. Statistical comparison involved the Wilcoxon Signed Rank Test. Fig. 2C: frequencies of HIV DNA harbouring cells assessed by a limiting cell dilution procedure (see Methods) in resting Tregs and non-Tregs from 12 patients. Statistical comparison involved the Wilcoxon Signed Rank Test. Fig. 2D: values of the percentages of HIV DNA harbouring cells in resting Tregs and resting non-Tregs (log_10_ scale) plotted against time under HAART. The equations of the tendency curves Y = Ae-^λt^ are indicated. The half life of latent Treg and non T reg reservoirs T_1/2_ (months) was determined as follows: ln2/λ.

We selected patients who were highly adherent to HAART and in whom plasma virus had been undetectable for 2 to 8 years with no reported plasma viral blips (Table 1). Within a pool of rigorously purified resting Tregs (CD25^hi^HLADR^−^CD69^−^CD4^+^ small size T cells) (>99% FoxP3^+^), we found the presence of infected cells at the cell number tested (Fig. 2A). We next examined the level of infection in Tregs, as compared to CD25^−^HLADR^−^ CD69^−^CD4^+^ small size T cells (quiescent non Tregs). We used real-time PCR to quantify HIV proviral DNA (see Methods). Resting Tregs showed a higher level of infection (p = 0.02) (Fig. 2B). We then assessed the frequency of infected cells in a series of 12 patients (Fig. 2C). We used a limiting cell-dilution PCR procedure (see Methods). In the Treg reservoir, the frequency of infected cells ranged from 1/1000 to 1/15000 cell equivalents (median 1/10000) but was significantly lower in the non-Treg reservoir, ranging from 1/5000 to 1/40000 (median 1/25000) (p = 0.002)(Fig. 2C). The frequency of HIV DNA harbouring cells in the Treg compartment ranged from 1.5- to 8-fold that of infected cells in the non-Treg subset. We determined the proportion of FoxP3^+^, CD25^+^FoxP3^+^ and CD25^hi^FoxP3^+^ cells in HLADR^−^CD4^+^ lymphocytes in a series of 49 patients including those tested for HIV DNA (Table 1). We can approximate that the CD25^hi^FoxP3^+^ reservoir is potentially ranging from 1.6% to 17% of the total resting CD4^+^ T cell reservoir. If one assumes that all FoxP3^+^ cells, regardless of CD25 expression, have the same frequency of infection as CD25^hi^FoxP3^+^ CD4^+^ T cells, one can estimate the relative size of the Treg reservoir as potentially ranging from 3.1 to 38.6% of the total resting CD4^+^ T cell reservoir.

**Table 1:** Immunovirological profiles of the study patients.

Number of patients	Time with undetectable plasma virus load (years)^1^	CD4+ T cell count at the time of study (cells/mm^3^)	FoxP3+ cells in HLADR-CD4+ T cells (%)	FoxP3+ CD25+cells in HLADR- CD4+ T cells (%)	FoxP3+ CD25hi cells in HLADR-CD4+ 3 T cells (%)
49	**5** [3–5.75] 2 (2–8)	**416** [328–556] (260–1300)	**4.6** [3.2–5] (2.1–7.3)	**3.3** [1.9–4.7] (2.6–3.5)	**1.6** [1.1–2.5] (1.4–1.9)

Table 1 reports immunovirological features of patients tested in virus detection and quantification experiments (experiments shown in Fig. 2A, 2B, 2C, 2D, Table 2 and supplementary Figure S1). Patients were on triple drug regimens which included 2 nucleoside analogues (Zidovudine, Lamuvidine, Stavudine , Didanosine, Abacavir, Emtricitabine or Tenofovir) in addition to one non- nucleoside reverse transcriptase inhibitor (Efavirenz or Nevirapine) or one protease inhibitor (Atazanavir, Lopinavir/ritonavir, Nelfinavir, Ritonavir or Saquinavir).

1 Limit of detection: 20 copies/ml.

2 Data are expressed as median[quartiles] and (range).

3 Working cell population for virological analysis.

In both Treg and non-Treg subsets, the frequency of infected cells decreased according to time on HAART (R = −0.692 and −0.602, respectively, p<0.05, Spearman Test). Whatever the time on HAART, the frequency of HIV DNA-harbouring cells was higher in the Treg subset than in non-Tregs (Fig. 2C, 2D). We next examined a possible difference in the kinetics of decay of the Treg and non-Treg latent reservoirs. The estimated half-life of the Treg and non-Treg reservoirs was similar, at 20 and 23 months respectively (Fig. 2D). Taken together, our results show that 1- in patients on prolonged HAART , the Treg subset contains HIV-infected cells 2- HIV DNA harbouring cells appear more abundant in the Treg subset than in non-Tregs but both virus reservoirs have a similar half-life.

### Infected Tregs could release virus in activation conditions that disrupt their quiescence

We next examined whether virus contained in resting Tregs could be recovered following appropriate cell activation. Previous studies have shown that following strong in vitro cell activation (PHA+IL-2+cocculture with allogeneic CD8-depleted PBMC) only 1% of the HIV-1 DNA-containing resting CD4^+^ T cells could be induced to transcribe HIV-1 and likely less are capable to release virus [26]–[28]. The median frequency of HIV DNA-harbouring cells in Tregs was 1/10000. However, we were able to purify only 300 000 to 400 000 resting Tregs from 30 ml blood samples, as resting Tregs represented from 2 to 7% of the HLADR^−^ CD4^+^ T cell subset (see above). Cells were treated with PHA+IL-2, a condition that permitted detectable Treg proliferation (Fig. 1D). We used 150 000 to 200 000 cells in both the untreated control well and the PHA+IL-2 activated well. Direct coculture of Tregs with allogeneic CD8-depleted HIV negative PBMC was not used, due to the suppressive effects of Tregs on proliferation of conventional CD4 T cells (Fig. 1C). As shown in Table 2, no spontaneous virus release was detected in resting Tregs, despite the use of a sensitive RT-PCR (limit of detection of 20 copies/ml). This control indicated the non-productive state of cell infection in resting Tregs. Following 3 weeks cell activation, HIV RNA production was found in cell culture supernatants in 7 out of 21 patients tested (33%) (Table 2).

**Table 2:** Infected Tregs could release virus in activation conditions that disrupt their quiescence.

	Untreated resting Tregs	Resting Tregs PHA+IL-2	Untreated resting non-Tregs	Resting non-Tregs PHA+IL-2
200 000 purified cells				
Patients with virus producing cells (limit of detection = 20 copies/ml)	0/21	7/21	0/23	3/23
HIV RNA (log_10_copies/ml) in positive supernatants (mean±sem)	-	3.27±1.48	-	2.70±0.52

150 000 to 200 000 resting Tregs (CD25^hi^HLADR^−^ CD69^−^CD4^+^ small size T cells) (>99% FoxP3^+^) were activated with PHA+IL-2 or left untreated (controls). Resting non-Tregs (CD25^−^HLADR^−^ CD69^−^CD4^+^ small size T cells) were tested at the exact cell number of 200 000/well. At day 21 of culture, supernatants were assayed for HIV RNA by quantitative RT-PCR (limit of detection of 20 copies/ml).

We could re-obtain blood samples in 4 patients in whom HIV RNA production could be detected from 200 000 Tregs. Cells were activated as above but supernatants instead of RT-PCR testing were used to infect PHA+IL-2 activated allogeneic CD8-depleted PBMC from HIV seronegative subjects (see methods). High amounts of HIV P24 were found in supernatants (>400 pg/ml) for patients tested, indicating effective virus amplification on allogeneic PBMC. As controls, we also tested in the same conditions of cell number (200 000 cells) and cell activation, resting non-Tregs. Viral RNA production was detected following PHA+IL-2 activation of 200 000 resting non-Tregs in few patients (3 out of 23 patients) (Table 2). Viral amplification following addition of supernatants on PHA+IL-2 activated allogeneic CD8-depleted HIV negative PBMC could be obtained in 2 out of 3 patients (HIV P24>400 pg/ml). One patient was negative despite detectable HIV RNA in initial cell culture supernatant. Overall these results suggest that infected Tregs could release virus in activation conditions that disrupt their quiescence and anergy.

Of interest, recent reports suggested that histone deacetylase inhibitors could lead to virus expression without full cell activation [29]. We tested the effect of valproic acid on 150 000 to 200 000 quiescent Tregs from patients under long-term HAART. Cells were activated for a period of 7 days. No viral RNA was detected in untreated wells (not shown). No significant expression of the activation marker HLADR was found following cell treatment with valproic acid for 7 days (not shown). In the patients tested, valproic acid led to very low but detectable virus production (see methods) in a sensitive quantitative RT-PCR (detection limit, 20 viral RNA copies/ml) in 5 of 10 patients tested (log_10_ copies/ml in HIV RNA positive supernatant: 1.46±0.16, mean±sem) and without detectable cell proliferation (not shown). The low level of viral RNA in supernatants, likely, was related to the limited amplification of virus produced by the rare in vivo infected cells, due to the limited effects of valproic acid on activation of uninfected cells. However, the keypoint is that valproic acid can potentially lead to virus expression in latently infected Tregs, therefore making them visible for cytotoxic CD8 T cells, through formation of MHC class I-virus peptide complexes.

### Targeting the Treg reservoir: specific constraints

**Figure 3:**
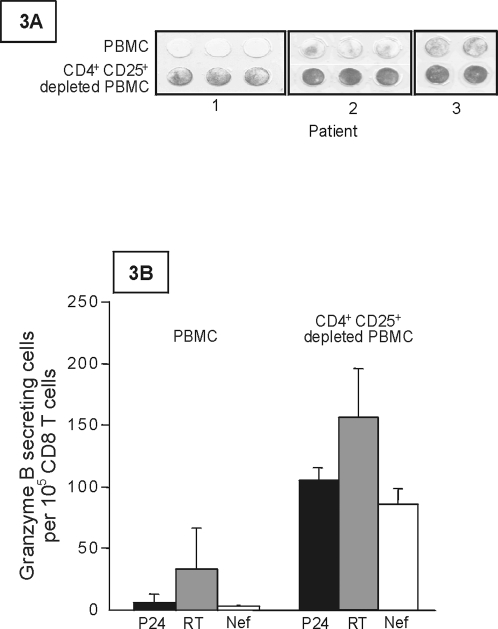
Activated Tregs inhibit granzyme B secretion by CD8 T cells. PBMC and CD4^+^ CD25^+^ cell-depleted PBMCs were assayed in three patients by ELISPOT for granzyme B secretion, following activation with PMA-ionomycin (Fig. 3A) or with overlapping HIV peptide pools corresponding to the p24, reverse transcriptase and Nef regions (Fig. 3B). Results shown in Fig. 3B are numbers of spots per 100 000 CD8^+^ T cells. Results correspond to the mean±SEM of results obtained in 4 patients.

**Figure 4:**
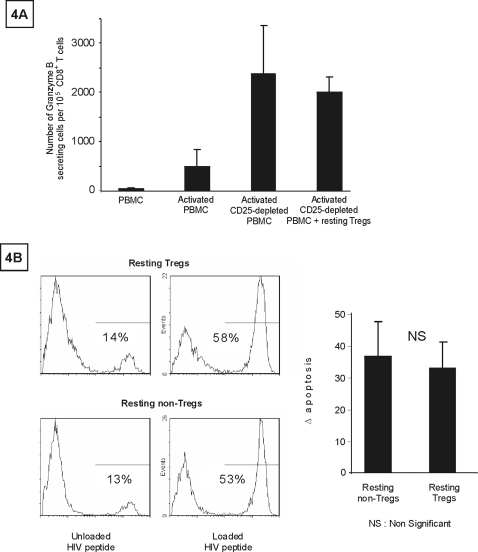
Quiescent Tregs are sensitive to specific CD8^+^ T cell cytotoxicity. Fig. 4A: CD25^+^ cell-depleted PBMCs were activated with PMA-inonomycin then extensively washed. Untreated HLADR^−^CD25^+^CD4^+^ T cells were then added at a physiological ratio. Cells were assayed by ELISPOT for granzyme B. Results correspond to the mean±SEM of results obtained in 2 patients. In Fig. 4B, HIV-specific CTL were co-cultured with CD25^+^HLADR^−^CD4^+^ and CD25^−^HLADR^−^CD4^+^ T cells loaded with HIV peptides. Controls were co-cultures of CD4^+^ T-cell subsets without peptides. Apoptosis was analyzed following annexin V staining on CD25^hi^HLADR^−^CD4^+^ and CD25^−^HLADR^−^CD4^+^ T cells. Results are expressed as the difference in the percentage of annexin V-positive cells in the presence and absence of HIV peptides. Results correspond to the mean±SEM of results obtained for 7 patients. Statistical comparison involved the Wilcoxon Signed Rank Test.

Approaches based on cell activation have been proposed to diminish or eliminate the HIV lymphocyte reservoir in patients on prolonged HAART. However, in addition to the hyporesponsiveness of Tregs, which may limit cell activation and virus expression, activated Tregs exert suppressive effects on both CD4^+^ and CD8^+^ T cells [20], [21], [30], [31]. Granzyme B is directly involved in CTL-mediated killing, and we found that CD25^+^CD4^+^ T-cell depletion strongly increased granzyme B release by CD8^+^ T cells following nonspecific activation with PMA+ionomycin (Fig. 3A). Depletion of CD25^+^CD4^+^ T cells also significantly increased granzyme B secretion in response to 15-mer overlapping HIV peptide pools corresponding to the RT, P24, and Nef gene products (Fig. 3B). These peptides could have activated specific Tregs, which then exerted their suppressive effects. Alternatively, this suppressive effect may be related to the existence, among untreated peripheral blood mononuclear cells (PBMCs), of already activated Tregs that could be continuously stimulated through interaction with self peptides. This latter possibility may explain the high proportion of HLADR^+^ cells among Tregs shown by ex vivo flow cytometry analysis (the median percentage of HLADR^+^ cells among CD25^hi^FoxP3^+^ CD4^+^ T cells was 51%, quartiles [46–59%], n = 15 patients on HAART for at least 2 years) (not shown). Activated Tregs might therefore create an environment in which CD8^+^ T cells are unable to fully exert their cytotoxic functions. In contrast, resting Tregs (HLADR^−^ Tregs without exogenous activation) have no significant inhibitory effects on granzyme B secretion by activated CD8^+^ T cells (Fig. 4A). Histone deacetylase inhibitors could lead to virus expression without full cell activation [29] (see above). This could be an approach of potential interest for reducing the resting Treg reservoir. We next examined whether resting Tregs but expressing HIV peptides were susceptible to CD8^+^ T-cell cytolysis. Owing to the low frequency of infected cells and specific CD8^+^ T cells, we used the following strategy. CD4^+^-depleted PBMCs were first screened for reactivity to several pools of overlapping peptides corresponding to the Gag, Pol, and Nef sequences (see Methods). The most reactive overlapping peptide pool was then used to amplify specific CTL for testing against peptide-loaded autologous resting Tregs and resting non-Tregs (see methods). As shown in Fig. 4B, similar levels of apoptosis were found in the Treg and non-Treg subsets. This suggested that resting Tregs expressing HIV peptides may be as susceptible as non-Tregs to CD8^+^ T-cell cytotoxicity.

## Discussion

In this study we identified a virus reservoir among resting regulatory CD4^+^ T cells in patients on long-term effective HAART. The frequency of HIV DNA harbouring cells was higher in the resting Treg subset as compared to the resting non Treg subset. HIV productive cycle occurs preferentially in activated cells. In vitro, Tregs are anergic or hyporesponsive as compared with conventional CD4^+^ T cells. Tregs share several biochemical features with anergic cells [32]. However, several studies in mouse models suggest that, in vivo, at least some Tregs can be activated to proliferate as strongly as conventional CD4^+^ T cells [33], possibly because the higher levels of IL-2 available in vivo are able to reverse the previously mentioned biochemical defects [32]. However, impaired IL-2 production during active HIV infection [34], [35] could cause Tregs to behave in vivo as in vitro. Such Treg hyporesponsiveness may potentially favour blockades in the viral cycle at the integration phase, leading to post-integration viral latency (see supplementary Figure S1). Moreover, FoxP3 which is expressed in Tregs was recently shown to inhibit HIV-1 LTR activation by targeting the NFκB pathway [36]. We also found that resting Tregs were intrinsically sensitive to specific CD8^+^ T cell-mediated cytotoxicity. However, upon activation, these cells inhibited CD8^+^ T-cell cytotoxic-associated functions such as granzyme B secretion. By helping Tregs escape from CTLs, this process could favour the survival of productively infected activated Tregs, allowing them to return to a quiescent state. HAART may promote the persistence of HIV or opportunistic agent-specific Tregs in the resting state, as the likelihood of re-encountering their specific antigens becomes very low.

The decrease in the size of the Treg reservoir in patients on HAART was similar to that of non-Tregs. This suggested that the factors leading to a higher level of infection in the Treg subset as compared with non-Tregs mainly act by favouring Treg reservoir formation, instead of slowing its decay. However, the process could be more complex: the apparent half-lives of the Treg and non-Treg reservoirs could be the result of additive, distinct and even antagonistic phenomena, including infected-cell decay in the two subsets, as well as virus replenishment through residual replication [37], [38]. Ramratnam et al. recently reported a shorter half-life of the latent reservoir in patients on intensified HAART (which may reduce residual viral replication) than in patients on standard HAART [38].

It has been shown in patients on long-term HAART that activated CD4^+^ T cells harbour actively replicating virus and, ex vivo, release virus spontaneously. Such ongoing virus replication, despite HAART, may continuously replenish the latent reservoir [28].

The half-life of the latent Treg and non-Treg reservoirs was estimated at approximately 20 and 23 months, respectively. This half-life was shorter than the approximately 44 months for the whole latent reservoir previously reported [39], although it is of the same order of magnitude, indicating that eradication of this reservoir in patients on continuous and effective HAART would take several decades.

The time of HAART initiation during the course of infection could be of importance, as a recent study [40] showed a shorter half-life of the latent lymphocyte reservoir in patients who were placed on HAART very rapidly (less than 5 months after seroconversion) following the onset of symptoms of primary HIV infection. This was not the case of the patients in our study, who started HAART from approximatively 2 years following the diagnosis of infection.

In vitro activation conditions that partly overcome Treg anergy led to detectable production of virus from 200 000 cells in a significant proportion of patients tested, which suggests that Tregs could potentially release virus in vivo and contribute to the viral rebound observed upon HAART withdrawal. The question of how to purge this reservoir, should therefore be addressed. Specific constraints related to the biology of Tregs may limit the therapeutic options. Cell-activation-based approaches, with IL-2 or anti-CD3, have been proposed to purge the latent reservoir. Despite some early encouraging results, these approaches have not eradicated the virus [41]. The rationale was that cell activation may induce expression of the virus in latently infected cells, whereas HAART would avoid *de novo* cell infection. Thus, unveiled, latently infected cells could be targeted by cytotoxic T lymphocytes or destroyed by direct cytopathic effects [41], [42]. Whether IL-2 can directly activate Tregs in vivo remains to be determined. In vitro, in contrast to the combination of PHA+IL-2, IL-2 alone did not lead to detectable virus production by Tregs (not shown) or to significant proliferation. In cancer patients, IL-2 immunotherapy led to expansion of Tregs with potent suppressive activity in vitro [43]. Such an effect could be related to increased generation of Tregs in the thymus [44]. In the peripheral compartment, exogenous IL-2 may help to break virus latency only in Tregs that undergo physiological antigen stimulation, by favouring their proliferation. This effect may represent only a small fraction of latently infected resting Tregs. Moreover, activated Tregs may inhibit specific CD8^+^ T-cell cytotoxic functions. The addition of activating anti-CD3 to IL-2 might increase the activation of the latent Treg reservoir. However, in addition to adverse effects related to non-specific generalized immune activation, this approach would not overcome the inhibition of CD8^+^ T cell-mediated cytotoxicity by activated Tregs. The effect of IL-7 on virus reactivation in the latent Treg reservoir remains to be determined. In patients on HAART, this cytokine was effective at triggering HIV-1 reactivation in CD8-depleted PBMCs and CD25^−^HLADR^−^ resting CD4^+^ T lymphocytes [45]. Partial cell activation and proliferation could be one of the mechanisms by which IL-7 acts on virus latency [45]. Recent data show that IL-7 increases the survival of murine Tregs by inhibiting apoptosis but has no effect on their proliferation [46]. Moreover, in human Tregs, IL-7 receptor (CD127) is downregulated [24], [25] (see also Fig. 1A).

Histone deacetylase inhibitors such as valproic acid were recently shown to trigger virus expression in vitro in resting CD4^+^ T cells, without full cell activation [47], and to reduce the latent reservoir in vivo [29]. However, the hopes raised by this finding were recently tempered by the results of other studies showing no clear effect of valproic acid on the size of the latent reservoir in vivo [48], [49]. Further studies are needed to clarify the real impact of valproic acid. Nonetheless, such therapeutics directly targeting virus quiescence, instead of conventional approaches that aim to disrupt cell quiescence, appear, at least in theory, more appropriate to purge the Treg latent reservoir, because they could potentially bypass the barriers that protect HIV within this compartment (i.e., hyporesponsiveness and inhibition of CD8^+^ T cell functions upon activation). Indeed, quiescent Tregs expressing HIV peptides were as sensitive to CD8^+^ T-cell cytotoxicity as non-Tregs. We also found that valproic acid was able to induce detectable virus production by latently infected Tregs in vitro, warranting further investigations of the potential impact of histone deacetylase inhibitors on the latent Treg reservoir in vivo.

Together, these results identify Tregs as a particular compartment within the virus reservoir that may require a specific approach for its purging.

## Methods

### Patients and healthy donors

Patients with chronic HIV-1 infection were recruited on the basis of plasma viral load values <20 copies/ml (Amplicor Ultrasensible, Roche Diagnostics, Meylan, France) for at least 2 years without reported viral blips. All the subjects gave their written informed consent, and the study was approved by the local bioethics committee (CPP Bicêtre hospital).

### Treg isolation

PBMCs were isolated by use of Ficoll. Purified CD4^+^ T cells were obtained by negative selection with the CD4 T-cell isolation kit II (Miltenyi Biotec, Bergisch Gladbach, Germany). Highly purified (>99%) CD25^−^HLADR^−^ or CD25^−^HLADR^−^CD69^−^ CD4 T cells and CD25^hi^HLADR^−^ or CD25^hi^HLADR^−^ CD69^−^ CD4 small size T-cells were obtained by cell sorting (FacsAria, Beckton Dickinson, CA) or (MoFlo, DakoCytoformation, Carpinteria, CA). For each patient, the CD25^hi^ sorting gate in HLADR^−^ CD4^+^ small lymphocytes was pre-defined on the basis of Foxp3 expression. Briefly, a 4 color flow cytometry analysis of cell surface CD4, HLADR, and CD25 and intracellular FoxP3 was performed. This allowed to define a CD25^hi^ sorting gate in which CD4^+^HLADR^−^ small size lymphocytes are >99% FoxP3^+^ (1.1 to 2.5% of CD4^+^HLADR^−^ small size lymphocytes. Resting Tregs CD4^+^HLADR^−^(CD69^−^)CD25^hi^ were then sorted on this basis (see Fig. 1A). FoxP3 expression was checked again following cell sorting.

In the case of sorting with the MoFlo cell sorter, prior to cell sorting and in keeping with the biosafety guidelines, CD4^+^ T cells were fixed with 1% PFA (experiments shown in Fig. 2A). FacsAria sorting was carried out in a laminar flow hood, which allowed the sorting of live cells from HIV-infected cells. In the cell-proliferation experiments (Fig. 1D and 1E), that do not require a very high level of purity, we used a magnetic bead-based procedure to isolate Tregs: the Regulatory T Cell Isolation kit (Miltenyi Biotec) or the combination of a human CD4 T cell enrichment cocktail and a human CD25 selection kit (StemCell Technologies, Vancouver, Canada). Both procedures were modified by the addition of anti-HLADR-coated magnetic beads (HLA-DR microbeads, Miltenyi Biotec) to deplete HLADR^+^ cells. The isolation procedure was optimized to select high CD25-expressing cells, by reducing the usual concentration of anti-CD25 and magnetic beads by half. Purity of CD25^+^CD4^+^ T cells ranged from 85% to 93% (83–90% FoxP3 expression).

### Antibodies and flow cytometry

FoxP3 expression in CD25^+^CD4^+^ lymphocytes was analyzed by flow cytometry after cell-surface staining of Ficoll-isolated peripheral blood mononuclear cells (PBMCs) with anti-CD4-PC5 (Beckman Coulter, Villepinte, France), anti-CD25-PE (Miltenyi Biotec), and anti-HLADR-ECD (Beckman Coulter), followed by cell permeabilization (eBioscience FoxP3 Staining Buffer Set, San Diego, CA) and intracellular staining with anti-FoxP3-FITC (clone PCH 101; FITC anti-human FoxP3 Staining Set, eBioscience).

The following antibodies were used in 4-colour combinations for flow cytometric analysis and/or cell sorting of Tregs: anti-CD69-FITC (BD Pharmingen, San Diego, CA) or anti-CD69-APC (BD Pharmingen), anti-CD30-PE (Miltenyi Biotec), anti-CD127-PE (BD Pharmingen), anti-CD4-PC5 (Beckman Coulter) or anti-CD4-perCP (BD Pharmingen), anti-HLADR-ECD (Beckman Coulter) or anti-HLADR-FITC (BD Pharmingen), anti-CD25-PE (Miltenyi Biotec) or anti-CD25-APC (BD Pharmingen). Expression of CD154 (CD40 ligand) was examined intracellularly, to increase the sensitivity of detection, by staining with anti-CD154-APC (Miltenyi Biotec) after cell permeabilization (eBioscience).

### FoxP3 quantitative PCR

RNA was extracted with the Chomoczinsky method as modified in the RNABle® kit (Eurobio, Courtaboeuf, France), then reverse transcribed (1^st^ Strand cDNA Synthesis kit for RT-PCR, Roche Diagnostics). FoxP3 and GAPDH mRNA expression was quantified with a Light Cycler-based quantitative kinetic PCR (Roche). An external scale was used to quantify GAPDH and FoxP3 cDNA. To correct for variations in RNA recovery and reverse transcription yield, FoxP3 cDNA values were normalized to GAPDH cDNA values. The following primers were used: for FoxP3, 5′-agaggacttcctcaagca-3′ and 5′-gctgccagcagctacgatg-3′ and for GAPDH, 5′-ggtgaaggtcggagtcaacgga-3′ and 5′-gagggatctcgctcctggaaga-3′ (Proligo, Boulder, CO, USA). Gel and melting curve analysis of PCR products showed no primer dimers or non-specific PCR products that might interfere with quantitation. Controls containing no RNA and controls containing RNA but no reverse transcriptase were always used and were negative.

### Treg suppressive functions

To evaluate the suppressive properties of Tregs, sorted cells were activated for 5 days with plate-bound anti-CD3 mAb (100 µl of 5 µg/ml solution) (Immunotech, Marseille, France) in the absence or presence of non-Tregs at ratios ranging from 1/10 to 1/1. Cell subsets were sorted as described above. Cell proliferation was evaluated on day 5 by measuring thymidine incorporation. Fifty microliters of 1/50 [H^3^]thymidine solution (1 mCi/ml) (Amersham Pharmacia, Uppsala, Sweden) per well was added for the last 8 hours of culture. [H^3^]Thymidine incorporation was measured in a Micro Beta liquid scintillation counter (Micro Beta Counter Plus, Wallac, Turku, Finland), and results were expressed as the mean cpm of triplicate wells.

### HIV DNA analysis

To assess the frequency of infected cells among resting Tregs and non-Tregs, we used a limiting dilution PCR procedure. DNA was extracted from the same number of sorted quiescent Tregs and non-Tregs (100 000 to 200 000 cells) with the QIAamp®DNA Blood Mini Kit (Qiagen, Hilden, Germany) and recovered in 200 µl of sterile PCR-grade water. In all patients, to check that similar amounts of DNA were effectively recovered from the two cell subsets, GAPDH quantitative PCR was carried out on 2 µl of DNA extracted from both subsets by use of a Light Cycler procedure. If necessary, DNA concentrations were adjusted, then GAPDH quantitative PCR was performed again. DNA serial dilutions corresponding to 40 000 to 1 000 cell equivalents were then used per PCR. HIV PCR was performed in a 50 µl volume under the following conditions: 0.25 pM/µl of each primer, 2.5 mM MgCl2, 0.2 mM dNTPs, 1.5 U Taq Platinum, and buffer (Invitrogen, Carlsbad, CA). The following primers targeting the HIV V3 loop between the C2 and V4 regions of gp120 were used: 5′-acacatggaattaggccagt-3′ and 5′-ctgccacatgtttataatttg-3′ (MWG-Biotech AG, Ebersberg, Germany). The sensitivity of the PCR ranged from 1 to 5 attograms of purified specific PCR product, which suggested a sensitivity from 2 to 8 copies.

HIV DNA was quantified in sorted resting Tregs and non-Tregs by a real-time PCR procedure [50]. Results were expressed as normalized values (attograms of HIV DNA per 100 femtograms of GAPDH). In most cases, we obtained a significant amplification curve for the 1 attogram point of the external scale (made of serially diluted purified specific PCR product), which, according to the size of the PCR product, suggested a PCR sensitivity of at least 7 copies.

### Cell activation, virus production, and cytotoxicity

To assess the proliferative capacity of Tregs as compared to non-Tregs in response to stimuli, 100 000 cells from each subset were cultured with 1) plate-bound anti-CD3 mAb (100 µl of 5 µg/ml solution; Immunotech), 2) plate-bound anti-CD3 mAb+1 µg/ml soluble anti-CD28 mAb (Immunotech), 3) phytohemagglutinin (PHA 0.5 µg/ml; Sigma, St Louis, MO) and interleukin 2 (IL-2; 25 ng/ml, Roche) or 4) recall antigen- (CMV, PPD, TT) and HIV P24-loaded dendritic cells (see below). On days 3 and 7, cell proliferation was measured as described above. Results were expressed as the mean cpm of triplicate wells. Treg activation by antigen-presenting dendritic cells (DCs) was assessed as follows: monocytes were obtained from PBMCs by positive selection in MACS columns with microbeads conjugated to anti-CD14 antibodies (Miltenyi Biotec). Monocyte-derived DCs were obtained after 7 days of culture with GM-CSF (100 ng/ml) and IL-4 (100 ng/ml) (Peprotec, Rocky Hill, NJ). On day 7, the following recall antigens were added to the cultures for 1 day: tetanus toxoid (1 µg/ml, Statens Serum Institute, Copenhagen, Denmark), Purified Protein Derivative PPD (1 µg/ml, Pasteur-Mérieux), HIV P24 (5 µg/ml, Protein Science, Meriden, CT) and purified Cytomegalovirus CMV lysate (1 µg/ml, Biowittaker, Walkersville, MA). DCs were then matured with lipopolysaccharide (LPS; 1 µg/ml, Sigma) for 1 day before co-culture with CD25^+^HLADR^−^CD4^+^ and CD25^−^HLADR^−^CD4^+^ T lymphocytes for 7 days. On days 3 and 7, cell proliferation was assessed by measuring thymidine incorporation as described above.

For virus production, 150 000 to 200 000 resting Tregs and non-Tregs were left untreated or activated with PHA+IL-2 then cultured for 21 days. For virus production in the presence of valproic acid, 150 000 to 200 000 resting Tregs were cultured for 7 days with valproic acid (Sigma-Aldrich) at a final concentration of 1 mM. Supernatants were tested for HIV with the Amplicor Monitor assay (mean quantitation limit 20 copies/ml, Roche Diagnostics). In the case of cells activated with VA, the absolute number of HIV RNA in supernatants was very low from 1.34 to 1.7 log_10_ copies/ml. However, regardless of the absolute number of RNA copies (which depends on the OD values obtained with an internal standard), the presence of HIV RNA was considered positive or negative if the OD obtained for the HIV PCR product (following specific probe hybridization and the colorimetric procedure) was over or under 0.15, according to the manufacture's instructions. The ODs obtained for cell samples treated with VA ranged from 0.3 to 0.8. The ODs of untreated samples were under 0.15.

To assess the infectivity of the virus produced by latently infected Tregs, supernatants obtained from resting Tregs or non-Tregs activated with PHA+IL-2 as described above were used to inoculate 10^6^ CD8 T cell-depleted PBMCs obtained from HIV-seronegative patients. CD8-depleted PBMC pre-activated with PHA+IL-2 for 48 hours were inoculated with infectious supernatants by centrifugation at 2500 rpm for 1 hour. Cells were then cultured for 21 days in 10% fetal bovine serum-RPMI medium in the presence of IL-2 (25 ng/ml, Roche). Supernatants were then assayed for HIV P24 antigen by using two distinct ELISA tests (Vidas HIV P24 II, Biomerieux, Marcy l'étoile, France; and Innotest HIV antigen mAb, Innogenetics, Courtaboeuf, France), which gave similar results. All positive supernatants were >400 pg/ml (upper limit of linearity).

To assess granzyme B secretion by CD8^+^ T cells, we used the BD Elispot Human Granzyme B set (BD Bioscience) according to the manufacturer's instructions. Total PBMCs or PBMCs depleted of CD25^+^CD4^+^ cells (100 000 cells/well) were activated with PMA and ionomycin (final concentrations 50 and 500 ng/ml, respectively) or pooled HIV peptides (RT, Nef, Pol, Env, P17 and P24; final concentration, 2 µg/ml). In some experiments, CD25-depleted PBMCs were activated with PMA-ionomycin for 2 hours, washed extensively, and untreated HLADR^−^CD25^+^CD4^+^ cells, obtained by cell sorter, were added at physiological ratio (pre-determined by flow cytometry before cell separation). Spots were then counted with a Zeiss analyzer (Carl Zeiss Vision, Jena, Germany).

To examine Treg susceptibility to CTL, PBMCs were isolated from patients on prolonged HAART, 50×10^6^ PBMCs were frozen at −80°C, and 10×10^6^ fresh PBMCs were depleted of CD4 cells (CD4 Microbeads, Miltenyi). CD4-depleted PBMCs were subjected to IFN-γ ELISPOT by use of several pools of overlapping MHC class-I HIV peptides covering the regions Gag (11 pools of 11–15 peptides) (Protein Science), Pol and Nef (10 pools of 5–10 peptides) (Intracell, London, UK). Plates (Millipore, Billerica, MA) were coated overnight with an IFN-γ-specific capture antibody (mouse anti-human IFN-γ, clone 1-D1K, Mabtech, Nacka Strand, Sweden) at 1 µg/ml. HIV peptide pools were added (final concentration 2 µg/ml for each peptide) to CD4-depleted PBMCs (100 000 PBMC/well) and incubated at 37° C overnight. Mouse anti-human IFN-γ–biotin (final concentration 1 µg/ml, clone 7-B6-1 biotin, Mabtech) was then added. After overnight incubation at 4°C, the plates were incubated with Streptavidin–PAL (Extravidin-Alkaline Phosphatase, Sigma-Aldrich) and revealed with use of BCIP/NBT phosphatase substrate (Eurobio). Spots were counted as described above.

CD8^+^ T lymphocytes were then stimulated for 10 days with the most responsive peptide pool to obtain specific CTL. On day 10, the frozen PBMCs were thawed, and magnetic bead -purified CD25^+^HLADR^−^CD4^+^ and CD25^−^HLADR^−^CD4^+^ T cells were obtained as described above (Miltenyi Biotec). A total of 200 000 cells were then loaded overnight with the same HIV peptide pool used to obtain CTL and cocultured overnight with CTL (target/effector ratio 1/1). Apoptosis was then analyzed by flow cytometry (Beckman Coulter) after labelling with CD4-PC5 (Beckman Coulter), CD25-PE (Miltenyi Biotec), and Annexin V-FITC (BD Pharmingen). Annexin-V expression was analyzed on CD25^hi^HLADR^−^CD4^+^ and CD25^−^HLADR^−^CD4^+^ T cells.

### Statistical analysis

Statistical comparisons were based on the Wilcoxon signed rank test. Correlations between the frequencies of infected cells and the time on HAART were tested with Spearman's rank correlation test.

## Supporting Information

Figure S1 Detection of integrated virus in Tregs: To examine HIV integration, an Alu/LTR real-time nested PCR method [51] was applied to DNA extracted from resting Tregs (CD25^hi^ HLADR^−^ CD69^−^ CD4^+^ small size T cells) (>99% FoxP3+) and non-Tregs (CD25^−^ HLADR^−^ CD69^−^ CD4^+^ small size T cells). Five µl of DNA extract, corresponding to 50 000 highly purified resting Tregs was used. During the first round of PCR, the LTR primer can initiate the formation of a single-stranded DNA from both integrated and unintegrated HIV-1 DNA. To control for this asymmetric PCR, we performed nested PCR without Alu primers during the first-round PCR. In all patients tested, controls without Alu primers led to detectable PCR amplification. This prevented us for accurate quantification of integrated virus. PCR amplification with Alu primers yielded a stronger signal than that obtained without Alu primers, which qualitatively indicated the presence of integrated virus. Similar results of the presence of integrated HIV in Tregs were obtained in 3 other patients.
(4.04 MB TIF)
