# Supplementary figures and images for: Resting Regulatory CD4 T Cells: A Site of HIV Persistence in Patients on Long-Term Effective Antiretroviral Therapy
Source: PLoS One. 2008 Oct 1;3(10):e3305. doi: 10.1371/journal.pone.0003305 (PMC2551739; doi:10.1371/journal.pone.0003305)

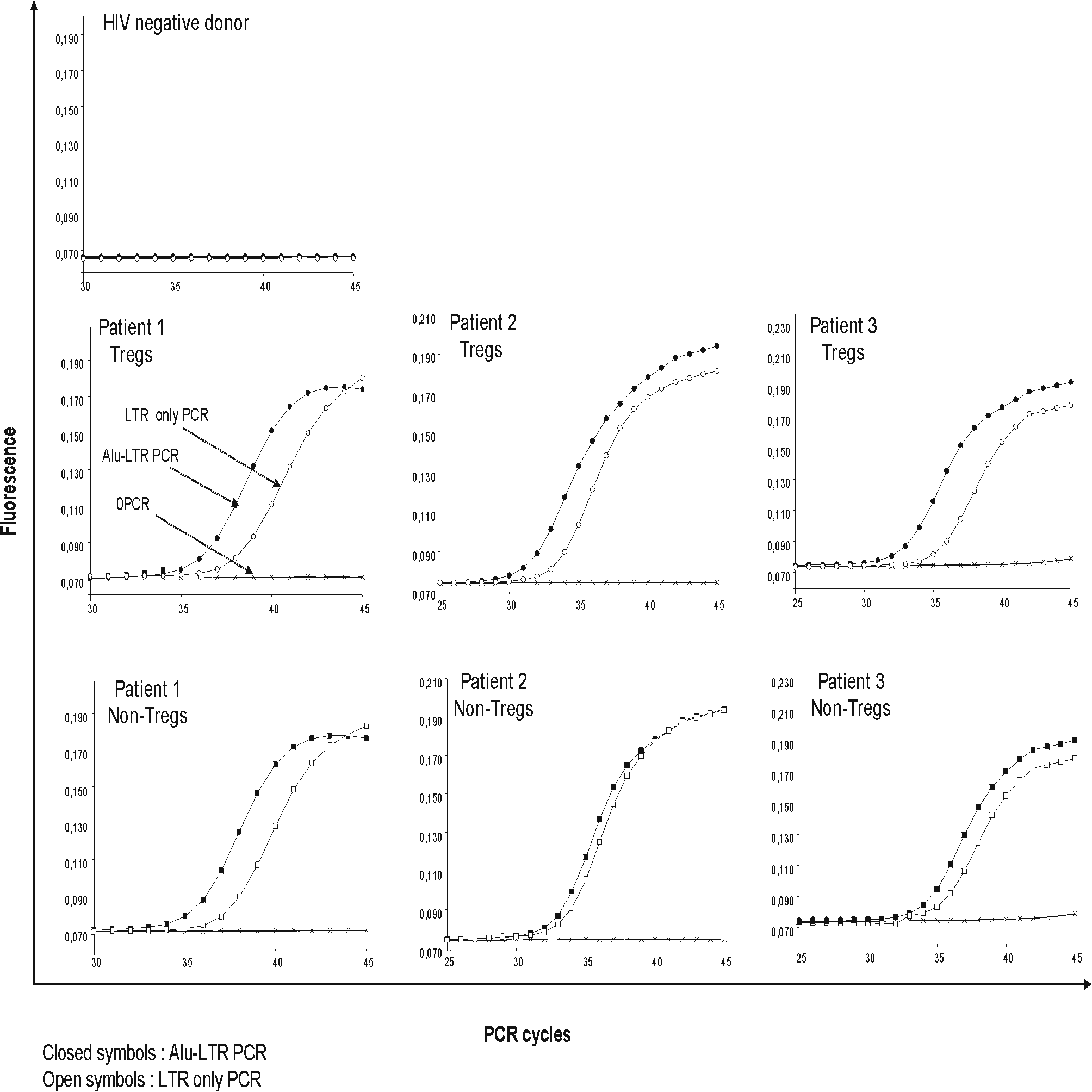

Supplement: Figure S1 — Detection of integrated virus in Tregs: To examine HIV integration, an Alu/LTR real-time nested PCR method [51] was applied to DNA extracted from resting Tregs (CD25hi HLADR− CD69− CD4+ small size T cells) (>99% FoxP3+) and non-Tregs (CD25− HLADR− CD69− CD4+ small size T cells). Five µl of DNA extract, corresponding to 50 000 highly purified resting Tregs was used. During the first round of PCR, the LTR primer can initiate the formation of a single-stranded DNA from both integrated and unintegrated HIV-1 DNA. To control for this asymmetric PCR, we performed nested PCR without Alu primers during the first-round PCR. In all patients tested, controls without Alu primers led to detectable PCR amplification. This prevented us for accurate quantification of integrated virus. PCR amplification with Alu primers yielded a stronger signal than that obtained without Alu primers, which qualitatively indicated the presence of integrated virus. Similar results of the presence of integrated HIV in Tregs were obtained in 3 other patients. (4.04 MB TIF) [file pone.0003305.s001.tif]
